# Major discrepancies between what clinical trial registries record and paediatric randomised controlled trials publish

**DOI:** 10.1186/s13063-016-1551-6

**Published:** 2016-09-23

**Authors:** Paola Rosati, Franz Porzsolt, Gabriella Ricciotti, Giuseppina Testa, Rita Inglese, Ferruccio Giustini, Ersilia Fiscarelli, Marco Zazza, Cecilia Carlino, Valerio Balassone, Roberto Fiorito, Roberto D’Amico

**Affiliations:** 1 G.A.L.I.L.E.O. Gruppo per l’Apprezzamento della Letteratura e l’Implementazione dei Livelli di Evidenza in Ospedale, Bambino Gesù Children’s Hospital IRCCS, Rome, 00165 Italy; 2 Unit of Clinical Epidemiology, Bambino Gesù Children’s Hospital IRCCS, Rome, 00165 Italy; 3 Health Care Research, General and Visceral Surgery, University Hospital Ulm, President Institute of Clinical Economics (ICE) e.V., Ulm, 89070 Germany; 4 Faculty of Medicine and Surgery, La Sapienza University, Rome, 00185 Italy; 5 General Surgery and Transplantation Department, University Tor Vergata, Rome, 00133 Italy; 6 Department of Diagnostic, Clinical and Public Health Medicine, University of Modena and Reggio Emilia, Modena, 41124 Italy

**Keywords:** Randomised controlled trials, Clinical trial registries, Reporting discrepancies, Selective outcome reporting, Quality reporting, Critical appraisal, Risk of bias, Dissemination bias, Reporting bias

## Abstract

**Background:**

Whether information from clinical trial registries (CTRs) and published randomised controlled trial (RCTs) differs remains unknown. Knowing more about discrepancies should alert those who rely on RCTs for medical decision-making to possible dissemination or reporting bias. To provide help in critically appraising research relevant for clinical practice we sought possible discrepancies between what CTRs record and paediatric RCTs actually publish. For this purpose, after identifying six reporting domains including funding, design, and outcomes, we collected data from 20 consecutive RCTs published in a widely read peer-reviewed paediatric journal and cross-checked reported features with those in the corresponding CTRs.

**Methods:**

We collected data for 20 unselected, consecutive paediatric RCTs published in a widely read peer-reviewed journal from July to November 2013. To assess discrepancies, two reviewers identified and scored six reporting domains: funding and conflict of interests; sample size, inclusion and exclusion criteria or crossover; primary and secondary outcomes, early study completion, and main outcome reporting. After applying the Critical Appraisal Skills Programme (CASP) checklist, five reviewer pairs cross-checked CTRs and matching RCTs, then mapped and coded the reporting domains and scored combined discrepancy as low, medium and high.

**Results:**

The 20 RCTs were registered in five different CTRs. Even though the 20 RCTs fulfilled the CASP general criteria for assessing internal validity, 19 clinical trials had medium or high combined discrepancy scores for what the 20 RCTs reported and the matched five CTRs stated. All 20 RCTs selectively reported or failed to report main outcomes, 9 had discrepancies in declaring sponsorship, 8 discrepancies in the sample size, 9 failed to respect inclusion or exclusion criteria, 11 downgraded or modified primary outcome or upgraded secondary outcomes, and 13 completed early without justification. The CTRs for seven trials failed to index automatically the URL address or the RCT reference, and for 12 recorded RCT details, but the authors failed to report the results.

**Conclusions:**

Major discrepancies between what CTRs record and paediatric RCTs publish raise concern about what clinical trials conclude. Our findings should make clinicians, who rely on RCT results for medical decision-making, aware of dissemination or reporting bias. Trialists need to bring CTR data and reported protocols into line with published data.

**Electronic supplementary material:**

The online version of this article (doi:10.1186/s13063-016-1551-6) contains supplementary material, which is available to authorized users.

## Background

An emerging problem that has rarely been investigated concerns the human factors that undermine clinical randomised controlled trials (RCTs) at various stages [1]. In the 1920s and 1930s two scientists in different research fields [2, 3] helped enormously to reappraise statistical theory and methodology in designing trials, thus clarifying how bias influences research. Previous papers have already compared protocols and registered data for clinical outcomes in published RCTs in various clinical settings [4–7], and three studies have investigated discrepancies in selectively reported outcomes [8, 9]. Even though concealment and blinding tools can control human factors in RCT designs [10] and the International Committee of Medical Journal Editors (ICMJE) [11] and Consolidated Standards of Reporting Trials (CONSORT) [12] receive wide consensus, a recent survey among journal editors endorsing the ICMJE and CONSORT established policies disclosed that only 27 % of the 33 respondents cross-checked the data reported in the submitted manuscript against the data registered prospectively in clinical trial registries (CTRs) [13]. In recent years the Food and Drug Administration (FDA) expanded the regulatory requirements for conducting clinical trials and the truthfulness of the data submitted, and now requires authors to annually update CTRs, thus providing clear informative results [14]. Whether trial design, conduction and outcome data from the various CTRs and published RCTs differ or are incompletely reported remains unknown [4, 15, 16]. One systematic review assessed primary outcome discrepancies [17]. No studies have analysed trial reporting domains more widely, and none have addressed paediatric trials. Apart from the Critical Appraisal Skills Programme (CASP) checklist [18], nor do paediatricians and clinical researchers have tools for assessing discrepancies and risk of bias that compare what clinical researchers record in the registered study hypothesis and protocol, and what they then publish in RCTs [19–23]. Knowing more about trial discrepancies should alert paediatricians, clinical researchers, peer-reviewers, editors, and policymakers to possible dissemination or reporting bias undermining paediatric trials whose results provide the best information for medical care [24].

To help in critically appraising research relevant for clinical practice we sought possible discrepancies between what CTRs record and paediatric RCTs actually publish. For this purpose, after identifying six reporting domains, including funding, design, and outcomes, we collected data from a sample of 20 unselected consecutive RCTs published in a widely read peer-reviewed paediatric journal and cross-checked reported features with those in the corresponding CTRs.

## Methods

In a study conducted from November 2012 to January 2016, to seek possible discrepancies between what CTRs record and paediatric RCTs actually publish, two reviewers, an experienced clinical paediatrician and an experienced researcher (PR and RD), identified six major reporting domains: five based on their long experience in critically appraising well-conducted clinical trials (reported funding and conflict of interest incompletely declared; discrepant or unclear sample size; inclusion and exclusion criteria not being respected or selective crossover; primary outcome downgraded and secondary outcomes upgraded and reported as primary outcomes in the publication; early study completion unjustified), and one domain (main outcome selectively reported or unreported) based on the Cochrane risk of bias tool [23, 24]. Over the first year they developed, assessed, and graded CTR-RCT discrepancy scores. Over the ensuing years five investigator pairs, supervised by two tutors (PR and RD), and attending the annual course held at Bambino Gesù Children’s Hospital, and subsequent weekly meetings on critically appraising scientific publications (G.A.L.I.L.E.O.), carefully read the 20 consecutive RCTs published monthly from July to November 2013 in the journal *Pediatrics* [25–44]. They then searched the CTR web link for each corresponding CTR, assessed details, and ‘history of changes’ after the initial registration, and critically appraised each published RCT with the CASP checklist. The five investigator pairs then independently mapped and coded inconsistencies for each reporting domain in the 20 trials, and repeatedly searched online (last access 20 January 2016) from the corresponding CTR websites: the United States National Institute of Health (NCT) (https://clinicaltrials.gov), the International Standard Randomised Controlled Trial Number (ISRCTN) (currently BioMed Central Open Access publishers) (http://www.isrctn.com/), the Nederlands Trials Register (NTR) (http://www.trialregister.nl/trialreg/index.asp), the Australian and New Zealand Clinical Trial Registry (ACTRN), (http://www.anzctr.org.au) and the Clinical Trial Registry-India (CTRI) (http://ctri.nic.in/Clinicaltrials/login.php). Meanwhile, PR and RD supervised the five investigator pairs for scoring the six individual reporting domains and combined scores. PR and RD then consulted the external tutor (FP), and conflicting interpretations were resolved by consensus. PR and RD with the five investigator pairs then reassessed inconsistencies between CTRs and RCTs for each trial over 2 months and, after several attempts, reached 100 % final agreement on grading discrepancy scores from 1 to 3 points, according to the reporting domain importance, and on grading combined discrepancy scores as low, medium and high (Table 1, Additional file 1). Higher discrepancy scores suggested risk of bias.

**Table 1:** Clinical trial registry (CTR)-randomised controlled trial (RCT) discrepancies in 20 RCTs scored by assessing and cross-checking inconsistencies in the six reporting domains identified, assessed and scored according to their importance in a well-conducted trial^a^

		Sponsors, funding and conflict of interests incompletely declared	Discrepant or unclear sample size	Inclusion/exclusion criteria not respected or selective crossover	Primary outcome or primary outcome measure modified or downgraded or secondary outcomes upgraded	Early RCT completion unjustified	Main outcome selectively reported or unreported	
		1	2		3			Combined discrepancy scores: low ≤4, medium 5–9, high 10–14^b^
CTR acronyms, registration numbers, and web link for the 20 RCTs published in the journal *Pediatrics* from July to November 2013 (first author and publication reference)	Cited references							
NCT 01351064, URL https://clinicaltrials.gov (Carroll et al. 132(3):e623-e 629 Sept 2013)	[25]	The RCT incompletely declared funding	-	-	-	-	The CTR automatically indexed the RCT reference but the authors failed to report the results Incongruities in the published RCT (abstract and results)	4
NCT 01822626, URL https://clinicaltrials.gov (Davoli et al. 132(5):e1236-e1245 Oct 2013	[26]	-	Number of eligible participants not provided in the CTR	-	-	-	The CTR automatically indexed the RCT reference but the authors failed to report the results	5
ISRCTN 59061709, URL http://www.isrctn.com/ (McCarthy et al. 132(2):e389-e395 Aug 2013)	[27]	-	-	The RCT failed to respect the exclusion criteria for three infants. During study conduction one infant crossed over	-	-	The CTR automatically indexed the RCT URL address but the authors failed to report the results	5
NTR 1613, URL www.trialregister.nl (van der Veek et al. 132(5):e1163-e1172 Nov 2013)	[28]	-	-	Discrepant children’s age in the inclusion criteria	-	-	The CTR failed to automatically index the RCT URL address or reference and the authors neglected to report the results. The published RCT reported that the intervention yielded insignificant results for all endpoints	5
ISRCTN 72635512, URL http://www.isrctn.com/ (Field et al. 132(5):e1247-e1256 Nov 2013)	[29]	-	-	-	-	Early study completion (March 2010 instead of May 2012)	The CTR automatically indexed the RCT URL address but the authors failed to report the results. The published RCT analysed selected data for randomised newborns in the intervention and control group (80 % vs 87 %) and reported an insignificant statistical difference for the primary outcome (cognitive improvement) but the authors inadequately reported more harm than benefit in the intervention group for secondary patient-centred outcomes (death, cerebral palsy)	6
NCT 00548379, URL https://clinicaltrials.gov (Aluisio et al. 132(4):e832-e840 Oct 2013)	[30]	-	-	Discrepant RCT inclusion criteria	Secondary outcome upgraded in the RCT	-	The CTR automatically indexed the RCT reference including the CTR primary outcome but neglected to report the RCT including the secondary outcome upgraded and showing an insignificant statistical difference between intervention and control group	8
ISRCTN 31707342, URL http://www.isrctn.com/ (McCarthy et al. 132(1): e135-e141 July 2013)	[31]	-	Discrepancy in the number of children enrolled and analysed (the CTR failed to report that an external Data Safety Monitor Committee stopped the RCT early hence the trial failed to reach the target number of participants)	-	-	The updated CTR failed to report early stopping after a planned interim analysis	The CTR automatically indexed the RCT URL address but failed to report early stopping. The RCT unclearly reported the reason for stopping the trial (more harm than good for the primary outcome in the intervention group)	8
NCT 01307293, URL https://clinicaltrials.gov (Shaw et al. 132(4):e886-e894 Oct 2013)	[32]	-	Discrepancy in the age of premature children enrolled	-	-	Early study completion (Dec 2012 instead of Jan 2013)	The CTR automatically indexed the RCT reference but the authors failed to report the results. The RCT reported benefits in the intervention group before study completion date	8
ACTRN 12608000056392, URL www.anzctr.org.au (Daniels 132(1):e109- e118 July 2013)	[33]	Funding incompletely declared in the RCT	Discrepancy in the number of participants eligible and enrolled	-	Primary outcome downgraded in the RCT	-	The CTR failed to automatically index RCT URL address or reference and the authors neglected to provide results The RCT failed to report that the intervention yielded insignificant results	9
NCT 00409448, URL https://clinicaltrials.gov (Kurowski et al. 132(1):e158-e166 Jul 2013)	[34]	Discrepancy in declared funding	-	Discrepant RCT exclusion criteria		Early study completion (Jan 2011 instead of August 2012)	The CTR failed to automatically index the RCT URL address or reference and the authors neglected to provide results	9
NCT 00551642, URL https://clinicaltrials.gov (Durrmeyer et al. 132(3):e695-e703 Sept 2013)	[35]	Funding incompletely declared in the CTR	-	-	Primary outcome completely changed in the RCT	Published ongoing study results and follow-up not respected	The CTR automatically indexed the RCT reference but the authors failed to report the results. The RCT neglected to report results for the primary outcome (more harm than good in the intervention group)	10
NCT 01403623, URL https://clinicaltrials.gov (Leadford et al. 132(1):e128-e134 Jul 2103)	[36]	Funding incompletely declared in the CTR	-	-	Primary outcome measure modified in the RCT	Early study completion (Oct 2011 instead of Dec 2012)	The CTR automatically indexed the RCT reference but the authors failed to report the results. Main outcome measure partially reported in the RCT	10
NCT 00334737, URL https://clinicaltrials.gov (Ohls et al. 132(1):e119-e127 Jul 2013)	[37]	Discrepancy in declared funding	-	-	Primary outcome partially modified in the RCT	Follow-up not respected (presumably an ongoing study or randomisation done for another study)	The CTR reported previous published authors’ papers (2004–2006) but failed to automatically index the RCT URL address or reference. The authors failed to report results in the last CTR updated in November 2013. The RCT only partially reported results on the primary outcome	10
NCT 01065272, URL https://clinicaltrials.gov (Alansari et al. 132(4):e810-e816 Sept 2013)	[38]	-	-	The RCT incompletely respected inclusion criteria	Primary outcome modified in the RCT	Early study completion (March 2012 instead of August 2012)	The CTR failed to automatically index RCT URL address or reference and the authors neglected to provide results. The RCT reported results for the modified primary outcome	11
NCT 01810978, URL https://clinicaltrials.gov (Dilli et al. 132(4):e932-e938 Oct 2013)	[39]	-	-	Discrepancy in the inclusion criteria	Primary outcome modified and secondary outcome upgraded in the RCT	Early study completion (Apr 2013 instead of May 2013)	The CTR automatically indexed the RCT reference but the authors failed to report the results. The RCT reported results for the modified primary outcome and upgraded secondary outcome	11
NTR 2061 and ACTRN 12610000230055, URLs www.trialregister.nl and www.anzctr.org.au (Kamlin et al. 132(2):e381-e388 Aug 2013)	[40]	Funding incompletely declared in the RCT	Discrepancy in the number of participants eligible and enrolled	Inclusion criteria modified in the Dutch register (NTR) only. The Australian registry (ANZCTR), retrospectively indexed failed to report inclusion criteria changes	-	Australian registry retrospectively indexed the trial and failed to report that the trial stopped early on an external data safety committee decision (difficulties in recruiting infants and futility)	The NTR, updated on 5 Apr 2012, failed to report completed results but stated that the trial was stopped for slow recruitment and futility. The ANZCTR was retrospectively registered and not updated (‘still recruiting’). The RCT abstract incompletely reported that the external data safety committee stopped the trial early owing to difficulties in recruiting infants. Data for 4 children excluded from the analysis in the RCT	11
CTRI 2010/091/001417, URL http://ctri.nic.in/Clinicaltrials/login.php (Malik et al. 132(1):e46-e52 July 2013)	[41]	The RCT failed to report that the trial was used for a medical thesis	Discrepancy in the number of participants eligible and enrolled	The RCT reported that to achieve the final sample size patients were enrolled from an area adjacent to the setting declared	Primary outcome downgraded in the RCT	-	The CTR, updated by the authors on 14 Jun 2012, reported a brief summary of positive results for the downgraded primary outcome but failed to report side effects. The RCT neglected to report increased side effects in the intervention group	11
ACTRN 12612000976886, URL www.anzctr.org.au (McIntosh et al. 132(2):326-331 Aug 2013)	[42]	-	The CTR neglected to report sample size	-	Primary outcome measure modified in the RCT	The CTR was retrospectively registered and the RCT failed to provide completion date	The retrospectively registered CTR failed to automatically index the RCT URL address or reference and the authors neglected to provide results. The RCT only partially reported results on the primary outcome	11
ISRCTN 03981121, URL http://www.isrctn.com/ (Wake et al. 132(4):e895-e904 Oct 2013)	[43]	-	Discrepancy in the number of participants eligible and enrolled	-	Primary outcome measure modified in the RCT	No study conduction period specified in the RCT. Results reported for only half of the declared follow-up	The CTR automatically indexed the URL address of the published RCT. The RCT neglected to report results for the primary outcome (no statistical difference) but reported a significant difference for a secondary outcome	11
NCT 01604460, URL https://clinicaltrials.gov (Belsches et al. 132(3):e656-e661 Sept 2013)	[44]	Discrepancy in declared funding	-	The RCT reported a modified intervention procedure for the included infants	Discrepancy in primary outcome measures	Early study completion (June 2012 instead of November 2012)	The CTR automatically indexed the URL address of the published RCT, but the authors failed to report results. The RCT only partially reported results on the primary outcome	12

^a^Reporting domains chosen from our clinical experience in critically appraising RCTs and the Cochrane Collaboration tool for assessing risk of bias (main outcome selectively reported or unreported) [23, 24]. Upgrading secondary outcomes means reporting secondary outcomes as primary outcomes in the publication. Discrepancies in the domain ‘main outcome selectively reported or unreported’ had similar scores because inconsistencies in this domain could have made the RCT results untrustworthy or less trustworthy. CTR abbreviations: the United States National Institute of Health (NCT), the International Standard Randomised Controlled Trial Number (currently BioMed Central Open Access publishers) (ISRCTN), the Nederlands Trials Register (NTR), the Australian and New Zealand Clinical Trial Registry (ACTRN), and the Clinical Trial Registry-India (CTRI). Trials are listed and grouped according to the combined discrepancy scores. When scores are identical, trials are listed alphabetically by first author surnames. ^b^Higher discrepancy scores suggest risk of bias

*CTR* clinical trial registry, *RCT* randomised controlled trial, *URL* Uniform Resource Locator

## Results

When we compared what the 20 paediatric RCTs published in the journal *Pediatrics* reported and what the collected five matching cross-checked CTRs recorded, 9 trials had medium (5–9) and 10 high (10–14) combined discrepancy scores (Table 1, Additional file 1). The five investigator pairs who critically appraised the trials with the CASP checklist found that all 20 published RCTs fulfilled the general criteria for assessing trial internal validity. Of the 20 RCTs, 11 were registered in the NCT [25, 26, 30, 32, 34–39, 44], 4 in the ISRCTN [27, 29, 31, 43], 2 in the ACTRN [33, 42], 1 in the NTR [28], 1 was registered both in the NTR and in the ACTRN [40], and 1 in the CTRI [41]. Assessment for the reporting domains disclosed that 9 trials had discrepancies in declaring sponsorship and conflict of interests [25, 33–37, 40, 41, 44], 8 trials had a discrepant or unclear sample size [26, 31–33, 40–43], 9 trials failed to respect inclusion or exclusion criteria [27, 28, 30, 34, 38–41, 44], 11 trials downgraded or modified primary outcome measures or upgraded secondary outcomes [30, 33, 35–39, 41–44], 13 trials completed early [29, 31, 32, 34–40, 42–44], and all 20 paediatric clinical trials selectively misreported outcomes or failed to report main outcomes, thus tending to overstress the positive results (Table 1, Additional file 1). A single-centre trial was retrospectively registered in the ACTRN [42], and one multicentre trial was registered prospectively in the NTR and retrospectively in the ACTRN [40]. For this multicentre trial, although the NTR reported that the trial had stopped, the ACTRN stated ‘still recruiting’, and neither CTR was updated. Two papers failed to respect the intention-to-treat analysis [29, 40]. Two trials were completed early during a planned interim analysis by an external Data Safety Monitoring Committee (DSMC): the first was stopped for efficacy results (more harm than good in the intervention group), and the target sample remained unreached, but the ISRCTN failed to report the cause [31], and the second, a multicentre trial, owing to futility in the results [40], underreported or misreported outcomes in the two registries (NTR and ACTRN). Published RCT abstracts and results both contained inconsistencies in reporting the reasons for stopping the trials [31, 40]. In another three CTRs and corresponding RCTs, the five investigator pairs detected a discrepancy between the primary outcome and efficacy results reported (more harm than good in the intervention group) [29, 35, 41]. Three published RCTs underreported insignificant results [28, 30, 33], one upgrading the secondary outcome [30], and one downgrading the primary outcome [33]. Two trials, one registered in the ACTRN and one in the CTRI, downgraded primary outcome in the published RCT [33, 41], two upgraded secondary outcomes, both registered in the NCT [30, 39], and another eight modified primary outcome or primary outcome measures, six registered in the NCT, one in ISRCTN, and one in ACTRN [35–39, 42–44]. For one clinical trial, the NCT automatically reported the previous RCT paper reference that included the NCT primary outcome, but neglected to report the RCT reference that included secondary outcomes that had been upgraded and yielded insignificant results [30]. For another clinical trial, the authors reported their previous published papers in the NCT record, but neglected to report the second published RCT giving a partially modified primary outcome in the updated NCT data, recorded after RCT publication [37]. Of the 20 clinical trials, seven CTRs (three registered in the NCT, two in the NTR, and two in the ACTRN) failed to index automatically the RCT reference or Uniform Resource Locator (URL) address, and all these trials had discrepancies in outcome data or yielded insignificant results [28, 33, 34, 37, 38, 40, 42]. Twelve CTRs reported the RCT references or URL addresses, but the authors failed to summarise the main results [25–27, 29–32, 35, 36, 39, 43, 44]. For only one clinical trial did the authors report the main results in the CTRI but neglect to report increased side effects in the intervention group [41] (Table 1, Additional file 1).

## Discussion

By comparing the six reporting domains, mapping, coding and cross-checking 20 published RCTs with the matched five CTRs, our study, applied to clinical paediatric trials published in a widely read peer-reviewed journal, suggests that many trials have discrepancies in reporting domains. The medium or high combined discrepancy scores we found, when we repeatedly searched each database online until January 2016, underline major widely ranging discrepancies between what CTRs record and what published paediatric RCTs then report. The discrepancies we identified in declaring funding and conflict of interests in nine trials, in the number of eligible and enrolled participants in eight clinical trials and in another nine inclusion and exclusion criteria not being respected, emphasise the generally imperfect reporting. These major discrepancies, especially those involving changes in the original study hypotheses, trial designs, study conduction and reporting outcomes raise concern on trustworthiness in scientific trials, as previous papers have underlined [5–9, 13, 15, 16].

Surprisingly, of the 20 published RCTs 11 modified or downgraded primary outcomes or upgraded secondary outcomes (reported secondary outcomes as primary outcomes in the publication), misreporting or tending to overstress positive results. This discrepancy underlines concerns about trial creditability that the CASP checklist overlooks. By assessing and scoring CTR-RCT discrepancies in six clinical trial reporting domains, our study therefore expands current knowledge, thus emphasising the need for international clinical trial regulators to make publicly available CTR-RCT discrepancies in published RCT findings, as recently underlined by the WHO Statement on public disclosure of clinical trial results (http://www.who.int/ictrp/results/reporting/en/; last accessed 20 January 2016). For example, trial (number 5 in Table 1) [29] addresses as primary outcome cognitive improvement and provides significant statistical difference between the intervention and control groups, but leaves unaddressed clinically important patient-centred outcomes, such as deaths and cerebral palsy. Even though deaths increased and cerebral palsy doubled in the intervention group, in their conclusions the investigators paradoxically report that ‘nonsignificant trends in the data suggested a small adverse effect’. In another trial (number 7 in Table 1) [31] an external DSMC decided to stop RCT completion early owing to hyperthermia in the infants enrolled in the intervention group. Neither the highlights section in the RCT nor the conclusions report this result. Although the published RCT reports when the trial stopped, our findings disclose an important clinically relevant feature, namely more harm than good for the primary outcome in the intervention group.

Another unexpected discrepancy in a multicentre trial (number 16 in the Table 1) [40] was that the authors inappropriately and unclearly reported that an external DSMC stopped the trial for futility. The numerous CTRs updated only by dataset supervisors (URL or RCTs reference cited in 12/20 trials), and failing to report results (7/20 trials) underline discrepancies involving incompletely and selectively reported clinical outcome results [5, 45–47]. This finding, along with underappreciated core patient-centred outcomes, raises ethical concerns and suggests dissemination or reporting bias [45–49]. Even though the journal *Pediatrics* complies with ICMJE requirements [11], and authors are required to submit a completed flowchart and checklist for the CONSORT statement [12] before publication (http://www.aappublications.org/content/pediatrics-author-guidelines#acceptance_criteria; last accessed 20 January 2016), and all the 20 published RCTs give the trial registration number on the first page, our findings underline that still today few authors endorse these rules [13, 14, 17].

An unexpected finding concerned prospective trial registration [11]. Although our study design did not require us to check clinical trial registration timing, we detected two trials (numbers 16 and 18 in Table 1) [40, 42] that had been registered retrospectively and failed to comply with ICMJE registration requirements. One of these two, a multicentre RCT (number 16 in Table 1) [40], was registered prospectively in the NTR and retrospectively in the ACTRN, and although the NTR reported that the trial had stopped for futility, the ACTRN stated ‘still recruiting’ Because a retrospectively registered trial could be hard to identify, regulators need to find new ways to encourage researchers to update information in a timely manner [50–52].

Most important, the major discrepancies our study highlighted in paediatric clinical trials give new clinically important information that researchers synthesising evidence from published RCTs in scientific literature reviews could fail to identify without cross-checking CTRs. Hence, they could provide less reliable scientific evidence, and misdirect future research priorities [49–53]. Our findings could also alert medical journals on the need to introduce rules that require investigators to submit the original Institutional Review Board-approved protocol (and its subsequent amended versions), and to explain later changes, thus helping reviewers and editors in deciding whether to publish the trial and what the final manuscript should state.

### Limitations

We acknowledge that our study has several limitations. Because we applied our study method only in few unselected consecutive paediatric RCTs published in a major paediatric journal, rather than including other authoritative journals with higher impact factors, our findings require further validation. Even though our scoring for reporting domains needs further refinement, our findings should make it easier for physicians to use research results from clinical trials. Because most authors neglected to update CTRs, we were unable to seek discrepancies in what authors recorded in CTRs and reported in published RCTs by cross-checking clinical outcome results. Similarly, because none of the five CTRs recorded or the cross-checked matched 20 RCTs appraised gave the necessary information, nor were we able to assess data for patient nonresponse and refusal. Although we analysed a small RCT sample, we found no differences in combined CTR-RCT discrepancy scores among the various CTRs. A final limitation is that we failed to assess interobserver reliability for the different assessor times for individual items and combined CTR-RCT discrepancy scores.

## Conclusions

Our study identifies major discrepancies between what CTRs record and paediatric RCTs publish. Our findings should make clinicians who rely on RCT results for medical decision-making, aware of dissemination or reporting bias. Trialists need to bring CTR data and reported protocols in line with published data. Clinical researchers and reviewers could search for CTR-RCT discrepancies to cross-check inconsistencies in core clinical trial reporting domains. Medical journals need to introduce rules that require investigators to submit the original Institutional Review Board-approved protocol (and its subsequent amended versions), and to explain any discrepancies. Assessing discrepancies would with little effort provide greater transparency, avoid wasting research resources, and encourage those who prepare medical recommendations and guidelines to think more critically. Future studies need to clarify whether the trial discrepancies we report warrant scepticism regarding study validity, or call for trialists to be more diligent about updating CTR data.

## Additional file

Additional file 1: Inconsistencies in the 20 trials published in the journal *Pediatrics* from July to November 2013, and discrepancy scores assessed by cross-checking what CTRs and their matching RCTs reported. Description of data: an operative table describing in detail the results of the 20 unselected consecutive RCTs, published in the journal *Pediatrics* from July to November 2013, mapped, coded, and cross-checked in six reporting domains to assess and report inconsistencies on what the authors recorded in CTRs and what they published in RCTs, as rated by predefined CTR-RCT discrepancy scores. (DOCX 56 kb)

## Abbreviations

ACTRN: The Australian and New Zealand Clinical Trial Registry
CASP: Critical Appraisal Skills Programme
CONSORT: Consolidated Standards of Reporting Trials
CTRI: The Clinical Trial Registry-India
CTRs: Clinical trial registries
ICMJE: International Committee of Medical Journal Editors
ISRCTN: International Standard Randomised Controlled Trial Number Registry
NCT: United States National Institute of Health Clinical Trial Registry
NTR: The Nederlands Trials Register
RCTs: Randomised controlled trials
URL: Uniform Resource Locator
