# Supplementary material for: Major discrepancies between what clinical trial registries record and paediatric randomised controlled trials publish
Source: Trials. 2016 Sep 23;17:430. doi: 10.1186/s13063-016-1551-6 (PMC5034459; doi:10.1186/s13063-016-1551-6)
Supplement: Additional file 1: — Inconsistencies in the 20 trials published in the journal Pediatrics from July to November 2013, and discrepancy scores assessed by cross-checking what CTRs and their matching RCTs reported. Description of data: an operative table describing in detail the results of the 20 unselected consecutive RCTs, published in the journal Pediatrics from July to November 2013, mapped, coded, and cross-checked in six reporting domains to assess and report inconsistencies on what the authors recorded in CTRs and what they published in RCTs, as rated by predefined CTR-RCT discrepancy scores. (DOCX 56 kb) [file 13063_2016_1551_MOESM1_ESM.docx]

**Additional file**

**Inconsistencies in the 20 trials published in the journal *Pediatrics* from July to November 2013, and discrepancy scores assessed by cross-checking what CTRs and their matching RCTs reported**

| **Reporting domains assessed** | **Sponsors, funding and conflict of interests** | **Sample size: number of patients (pts) eligible and enrolled.**  **Setting** | **Inclusion/exclusion criteria not respected or selective cross-over** | **Primary outcome or primary outcome measure and secondary outcomes** | **Study completion date** | **Main outcome selectively reported or unreported** |  |
| --- | --- | --- | --- | --- | --- | --- | --- |
| **Discrepancy scores** | 1 | 2 | 2 | 3 | 3 | 3 | **Combined discrepancy scores: low** ≤ **4, medium 5-9, high 10-14** |
| **Trial registry (CTR) numbers and web links for the 20 RCTs published (title, first author and reference details)** |  | | | | | | |
| 1. NCT01351064 https://clinicaltrials.gov (Use of a computerized decision aid for attention-deficit/hyperactivity disorder (ADHD) diagnosis: a randomized controlled trial. Carroll et al. 132, 3, e623-e 629 Sept 2013) | **The CTR declared funding from the National Library of Medicine (NLM) and Indiana University whereas the RCT declared funding only from the NLM** | The CTR failed to mention eligible pts. The RCT mentioned 183 eligible pts with ADHD and enrolled 84.  Setting: Four primary care practices. USA | Pts with ADHD randomised by ranking clinics, assigning 2 to the intervention and 2 to the control group.  *Appraisers raised doubts on the randomisation method chosen* | Identical primary and secondary outcomes in the CTR and RCT: expand and modify a computer-based decision support system (CHICA) to include ADHD guideline rules for diagnosis and treatment and also to improve patients functioning and adherence to recommendations | July 2012 | **The CTR automatically indexed the RCT reference but the authors failed to report the results.**  *The RCT abstract reported a significant difference between the intervention group (CHICA and ADHD guidelines) and control group whereas the results reported an insignificant difference* | 4 |
| 2. NCT 01822626 https://clinicaltrials.gov (Pediatrician-led motivational interviewing to treat overweight children: an RCT. Davoli et al.132, 5, e1236-e1245 Oct 2013) | Local Health Authority Reggio Emilia, Italy | **Number of eligible pts and sample size not provided in the CTR.**  **The RCT declared 419 pts as eligible. Participants were 372 overweight children 4-7 years with BMI at 85-95 percentiles.**  Setting: Reggio Emilia Province, Italy | Experimental motivational counselling versus usual care assessed by BMI control and subjective questionnaire answers  Overweight children 4-7 years with BMI at 85-95 percentiles  Excluded metabolic disorders | Identical primary and secondary outcome s in CTR and RCT: BMI variation from baseline at 12 months follow-up. Secondary outcomes: variation in physical activity and dietary behaviours at 12 months | Completion date respected (August 2012) | **The CTR automatically indexed the RCT reference but the authors failed to report the results.**  *Motivational interviewing (counselling) led to a statistically significant decrease in BMI in overweight children at 12 months follow-up. No difference versus usual care for less educated mothers* | 5 |
| 3. ISRCTN 59061709 www.controlled-trials.com (A randomized trial of nasal prong or face mask for respiratory support for preterm newborns. McCarthy et al.132, 2, e389-e395 Aug 2013) | National Children’s Research Centre | The CTR stated 142 < preterm (< 31 weeks) pts and the RCT 144 preterm participants requiring respiratory support in the delivery room (DR).  Setting: National Maternity Hospital Dublin, Ireland | **One infant crossed over to face mask (FM) from single nasal prongs (SNP) (intention-to-treat-analysis not respected?). Three infants (2 not requiring ventilation and 1 not requiring intubation) were included.**  *40 eligible preterm infants not enrolled because parents declined consent, or were not approached, or also if parents agreed were not enrolled* | Identical primary and secondary outcomes in the CTR and RCT: Intubation and mechanical ventilation with FM vs SNP. Secondary outcomes: heart-rate at 5 min, supplemental O2 in DR, compressions in DR, use of adrenaline and 23 other secondary outcomes | The CTR stated 30 June 2012. The RCT August 2012 | **The CTR automatically indexed the RCT URL address but the authors failed to report the results.**  *The RCT reported no difference in intubation rate between FM and SNP* | 5 |
| 4. NTR 1613 www.trialregister.nl (Cognitive behavior therapy for pediatric functional abdominal pain: a Randomized Controlled Trial. Van der Veek et al. 2013, 132:e1163-e1172) | Dutch Digestive Foundation (Maag Lever Darm Stichting) | Eligible pts not reported in the CTR but 201 reported in the RCT.  Setting: Emma Children’s Hosp. Acad. Medical outpatients’ clinic and De Bascule Ac Center Child Adolescent Psychiatry, Amsterdam-Netherlands | **The CTR stated as inclusion criteria children 8-18-years-aged with abdominal pain lasting 8 weeks or more (target number 100 pts. The RCT reported 104 children 7-18 yrs** | Identical primary and secondary outcomes in the CTR and RCT: Cognitive behaviour therapy (CBT) versus intensive medical care (IMC) to reduce abdominal pain. Secondary outcomes: anxiety, depression, somatic complaints, functional GI impairment, quality of life | The CTR declared the study completion date as 1 Nov 2010. The RCT stated completion in Aug 2011 | **The CTR failed to automatically index the RCT URL address or reference and the authors neglected to report the results.**  **The RCT reported insignificant results for CBT versus IMC for all endpoints.**  *Six pts dropped out in the CBT arm and 10 in the IMC arm. We hypothesise that researchers prolonged the planned closing date of the study owing to difficulties in recruitment. (One patient randomised to IMC asked to receive CBT and was considered dropped out and maintained in the IMC arm)* | 5 |
| 5. ISRCTN72635512 www.controlled-trials.com (Neonatal extra-corporeal membrane oxygenation (ECMO) study of temperature (NEST): a randomized controlled trial. Field 132, 5, e1247-e1256 Nov 2013) | British Heart Foundation | The CTR: failed to specify eligible newborns and stated a target number of 118 participants. The pragmatic RCT reported 118 eligible fully-grown newborns and 111 participants.  Setting: Four Neonatal ECMO centres, UK | Identical inclusion and exclusion criteria in the CTR and RCT | Identical primary outcome in the CTR and pragmatic RCT (intervention group ECMO plus cooling versus ECMO in control group to improve cognitive composite score) and secondary outcomes (death, cerebral palsy, language, seizures, visual impairment and other neurological signs or symptoms).  *Parents who completed self-report questionnaires at 2 years were aware about group allocation. Parents were asked not to reveal this information to the paediatrician who conducted the 2-year assessment* | **In the CTR completion date 31 May 2012. The pragmatic RCT stopped on 31 March 2010** | **The CTR automatically indexed the RCT URL address but the authors failed to report the results.**  **The RCT reported an insignificant statistical difference in cognitive improvements between the two groups, but did not clearly underline more harm than good in the intervention group for patient-centred outcomes (deaths increased and cerebral palsy doubled).**  *The RCT analysed data for only 80% of the randomised newborns in the intervention group and 87% in the control group (intention-to-treat analysis not respected). More deaths in the intervention than in the control group (9 vs 5)* | 6 |
| 6. NCT00548379 https://clinicaltrials.gov (Vitamin D_3_ supplementation and childhood diarrhea: a randomized controlled trial. Aluisio et al. 132,4, e832-e840 Oct 2013) | Wellcome Trust | Mild differences in the age and eligibility of pts enrolled (in the CTR 1-12 months and 3048 eligible pts; in the RCT 1-11 month-pts and 3060 eligible).  Setting: Five Kabul inner-city districts, Afghanistan | **The CTR included pts with pneumonia actively ascertained and passively surveyed through weekly home visits or from admission to the trial clinic in the study area.**  **The RCT included pts with diarrhoea, living in the study area, actively and passively surveyed with blood tests for level of vitamin D and recall from parents and caregivers during diarrhoea episodes** | **The CTR declared as primary outcome vitamin D supplementation for 3 months to reduce pneumonia in children aged 1-12 months (followed for 18 months) and secondary outcome incidence of diarrhoea and rickets.**  **The RCT reported as primary outcome vitamin D supplementation for 4 months to decrease recurrent diarrhoea in 1-11-month-old children followed for 18 months.**  *In the method section the authors specified that in the original protocol diarrhoeal illnesses were planned as a secondary a priori end-point* | June 2009 | **The CTR automatically reported the RCT paper reference including the CTR primary outcome but neglected to report the RCT including the secondary outcome upgraded (CTR record updated on Sept 2014).**  **The RCT reported the upgraded outcome result: no statistically significant difference in decreasing diarrhoea episodes by using vitamin D supplementation** | 8 |
| 7. ISRCTN 31707342 www.controlled-trials.com (A randomized trial of exothermic mattresses for preterm newborns in polyethylene bags. McCarthy et al. 132, 1, e135-e141 July 2013) | The National Children’s Research Centre Dublin, Ireland | **The CTR and RCT declared a target sample of 116 preterm infants whereas the RCT enrolled 93 and analysed data only for 72.**  **The CTR failed to report that the target sample was not reached because an external data safety monitoring committee (DSMC) stopped the RCT early.**  Setting: National Maternity Hospital Dublin, Ireland | The CTR stated known congenital anomaly with an open lesion such as gastroschisis or myelomeningocele as an exclusion criteria.  The RCT specified in the figure, one infant excluded because he was not resuscitated in the delivery room and no infants had congenital anomalies detected.  *The RCT specified randomisation for eligible newborns made before birth* | Identical primary and secondary outcome measures in CTR and RCT: rectal temperature 36.5-37.5°C. As secondary outcome measures they recorded ten other preterm variables | **The CTR stated 30.06.2012 as the end date (updated on 04.09.2013).**  **RCT stopped on Feb 2012 by the DSMC during a planned interim analysis** | **The CTR automatically reported the RCT URL address but failed to report early stopping.**  **The RCT publication reported in the results that the DSMC stopped recruitment because they identified more harm than good for the primary outcome in the intervention group, but neither the highlights nor the conclusions reported that the reason for stopping the trial early was hyperthermia in the intervention group** | 8 |
| 8. NCT01307293 https://clinicaltrials.gov (Prevention of traumatic stress in mothers with preterm infants: a randomized controlled trial. Shaw et al.132, 4, e886-e894 Oct 2013) | Stanford University | The CTR failed to describe eligible mothers and sample size. The RCT reported enroling 196 eligible and 105 >18-year-old-mothers of premature children at > 25-weeks-gestation with postpartum traumatic stress (PTSD) admitted to neonatal intensive care units (NICUs).  Setting: Four NICUs, California, USA | **The CTR stated >-18-year-old-mothers with PTSD and premature infants > 26 week-gestation and the RCT reported premature infants > 25 weeks-gestation** | Identical primary outcomes in CTR and RCT: 6-12 sessions of cognitive behavioural therapy (CBT) to reduce PTSD measured by Davidson Trauma Scale and BDI-II self-rating after 6-12 CBT sessions lasting 4-5 weeks | **Enrolment completed in December 2012 and follow-up completed in January 2013** (***did mothers complete 12 CBT sessions for the last infants enrolled?)*** | **The CTR automatically indexed the RCT reference but the authors failed to report the results.**  **The RCT reported a preliminary result (behavioural CBT significantly reduces PTSD at 4-5 weeks) before study completion date** | 8 |
| 9. ACTRN 12608000056392 www.anzctr.org.au (Outcomes of an early feeding practices intervention to prevent childhood obesity. Daniels 132, 1, e109- e118 July 2013) | **The CTR declared 8 sponsors whereas the RCT reported only 4 sponsors and some of the sponsors differed from those reported in the CTR** | Small differences in the sample size (in the CTR 830 and in the RCT 839) of full term and normal weight 4-7 month aged infants delivered by > 18-years-first-time-mothers.  Setting: two Australian cities (Brisbane and Adelaide) | **The CTR specified that this multicentre study would include infants born in two towns (Brisbane and Adelaide) and 10 hospitals or institutions. The RCT reported that the trial would include infants from two towns (Brisbane and Adelaide) and 7 hospitals or institutions** | **Primary outcome in the CTR: infant food intake preference and feeding behaviour after receiving anticipatory guidance on early feeding practices vs standard care by measuring 3 non-consecutive days using a 2 x 24 hour record and 1 x 24 hour recall after 2 consecutive 9-month modules. The RCT defined the intervention as interactive group sessions delivered when children were 4 months and 20-months old. The RCT alone specified lower anthropometric indicators of obesity risk at 2 yrs of age as a secondary outcome** | The CTR declared enrolment by 19.08.2009 and 18 months follow-up. The RCT gives the results gathered over 6-8 months after the total planned intervention completed when children were 2 yrs old | **The CTR failed to automatically index RCT URL address or reference and the authors neglected to provide results.**  **The RCT failed to report results for primary outcome but reported finding no statistically significant differences in anthropometric outcomes or in prevalence of overweight/ obesity even though mothers in the intervention group reported early feeding practice more frequently than those in the control group.**  *1/3 lost to follow-up in the intervention group vs control group (92/65 pts).* | 9 |
| 10. NCT 00409448 https://clinicaltrials.gov (Online problem-solving therapy for executive dysfunction after child traumatic brain injury. Kurowski et al. 132, 1, e158-e166 Jul 2013) | **The CTR declared funding from Children’s Hospital Medical Center, Cincinnati.**  **The RCT reported Colorado Traumatic Brain Injury Trust Fund Research Program and the NIH** | Teens who sustained a traumatic brain injury. Eligible 308, randomised 132 and analysed 120.  Setting: Five tertiary hospitals, USA | **Exclusion criteria in CTR: children previously suffering from psychiatric problems or mental retardation, autism or traumatized by child abuse or suffered a non-blunt injury (such as a projectile wound, stroke) or not living with parents or guardian.**  **The RCT also added as an exclusion criteria insufficient recovery to allow participation in the study or parental psychiatric hospitalization within 1 year before enrolment** | Identical primary and secondary outcomes in CTR and RCT: parent and teen self-reported-measures on improving the Internet-based counselor-assisted problem solving (CAPS) vs CAPS plus a trained counselor visiting the families through a 6-month program and one-on-one videoconference sessions (CAPS intervention) in teenage children who sustained in the previous 6 months a traumatic brain injury | **In the CTR August 2012.**  **In the RCT Jan 2011.**  *The RCT publication reported on the first page that dr Kurowski interrupted the analyses but did not provide the reason* | **The CTR failed to automatically index the RCT URL address or reference and the authors neglected to provide results.**  *The RCT reported significant improvement in executive functional behaviours at 6-month follow-up in older teens in the CAPS group compared with the internet-resource-comparison group. No further differences were found* | 9 |
| 11. NCT 00551642 https://clinicaltrials.gov (Two-year outcomes of a randomized controlled trial of inhaled nitric oxide in premature infants. Durrmeyer et al. 132,3, e695-e703 Sept 2013) | **INO therapeutics in the CTR.**  **In the RCT, INO therapeutics and other funding and supports for the authors’ travel and consulting fees** | The CTR and RCT declared 800 eligible preterm infants (24-29 weeks gestation) developing respiratory distress syndrome and requiring surfactant and continuous positive airway pressure (CPAP).  Setting:: US, UK, Germany, France, Italy, Spain, Belgium, Finland, Sweden | Identical inclusion and exclusion criteria in the CTR and RCT declared: preterm infants within 24 hours after birth with signs of respiratory distress and excluded infants born at > 29 gestational age; requiring high oxygen pressure, suspected heart disease, abnormal homeostasis, other anomalies or medications | **The CTR purpose declared was to evaluate safety and efficacy of inhaled nitric oxide (INO) vs placebo to reduce the risk of chronic lung disease in preterm infants with respiratory distress and to assess the long-term effects of the therapy on the development of these children at 7 years follow up.**  **The RCT reported as main outcome neurodevelopment at 1 and 2 years of age and respiratory and other outcomes in survivors** | **The CTR declared study completion on March 2015.**  **The RCT reported that this is an ongoing study for which at 7 years additional neurologic assessment will be performed** | **The CTR automatically indexed the RCT reference but the authors failed to report the results.**  **The RCT reported results on modified primary outcome and failed to report results on survival in conclusions (57 infants died with INO and 50 died with placebo). Differences in neurodevelopment between the two groups at 2 years not reported** | 10 |
| 12. NCT 01403623 https://clinicaltrials.gov (Plastic bags for prevention of hypothermia in preterm and low birth weight infants. Leadford et al. 132, 1, e128-e134 Jul 2013) | **The CTR declared funding from University of Alabama of Birmingham US. The RCT reported other grants (Eunice Kennedy Shriver, National Institute of Child Health, other Networks, National Institute of Health (NIH)** | The CTR and RCT reported the same sample size: 106 preterm infants with estimated gestational age between 26-36.6 weeks or with expected birth weight 1000-2500 g enrolled and randomised within the first 10 min after birth in the delivery room.  Setting: Zambia, Africa.  *Not cited approval from the institutional review board in Zambia* | Identical inclusion criteria in the CTR and RCT.  Exclusion criteria declared only in RCT (malformation and skin disease) | **The CTR stated primary outcome measure: axillary temperature taken per axilla for 1 min, 36.5-37.5 degrees Celsius (time-frame 1-4 hours).**  **The RCT reported the same axillary temperature but failed to specify minutes for measuring temperature** | **The CTR declared completion date on December 2012.**  **The RCT reported Oct 2011** | **The CTR automatically indexed the RCT reference but the authors failed to report the results.**  **The RCT reported statistically significant results between those using plastic bag and routine practice at 1 hour, but failed to report results at 4 hours.**  *Authors neglected to report two hyperthermic infants in the intervention group (temperature over 37.5°C)* | 10 |
| 13. NCT 00334737 https://clinicaltrials.gov (A randomized, masked, placebo-controlled study of darbepoetin alfa in preterm infants. Ohls et al. 132,1, e119-e127 Jul 2013) | **The CTR declared funding from University of New Mexico, Thrasher Research Fund and University Colorado.**  **The RCT declared the same funding source and 3 other sponsorships** | The CTR and RCT declared same sample size 102 preterm infants (≤32 weeks-gestational age and less than 2 days of age in a three- arm study between darbepoetin, erythropoietin and placebo (sham injection).  Setting: Four hospitals in Colorado, New Mexico and Utah (high-altitude centers) | The CTR and RCT reported the same criteria for inclusion and exclusion | **The CTR reported as primary outcome number of transfusions during hospitalization and composite cognitive score at 18-22 months corrected age and as secondary outcome haematocrit, reticulocyte count, overall neurodevelopment impairment, incidence of retinopathy of prematurity stage 3 or greater, anthropometric measures.**  **The RCT reported as primary outcome increased reticulocyte counts, decreased number of transfusions, and mental developmental index at 18-22 months.** | **The CTR declared study completion in June 2014.**  **The RCT reported May 2010** | **The CTR reported previous published authors’ papers (2004-2006) but failed to automatically index the RCT URL address or reference. The Authors failed to report results in the last CTR updated in November 2013.**  **The RCT reported reduced transfusion in the groups treated with darbepoetin and erythropoietin compared with placebo but failed to report similar effect between darbepoetin and erythropoietin groups and results on composite cognitive score.**  *This is probably an ongoing study or randomisation done for another study* | 10 |

| 14. NCT 01065272 https://clinicaltrials.gov (Oral dexamethasone for bronchiolitis: a randomized trial. Alansari et al. 132,4, e810-e816 Sept 2013) | The CTR and RCT declared Hamad Medical Corporation | The CTR declared a same sample size of 200 eligible infants 1 m-<18 months with bronchiolitis with high severity score ≥ 4 with and a familiar history for eczema or asthma (parent or full sibling).  The RCT: reported 102/98 participants each arm.  Setting: Paediatric Emergency Centre, Al-Saad, Quatar | **Identical inclusion criteria in the CTR and RCT: infants <18 months with severe bronchiolitis and parents or sibling with a history of asthma.**  **The CTR failed to declare exclusion criteria. The RCT excluded, premature or previously wheezing infants, critically ill infants, and infants with congenital heart defects and cystic fibrosis** | **The CTR primary outcome declared was infants’ discharge at 12 hours (intervention dexamethasone plus salbutamol vs salbutamol plus placebo). Secondary outcomes discharge at 18, 24, 36 and 48 hours.**  **The RCT reported primary outcome modified as geometric mean duration until readiness for discharge** | **The CTR completion date was August 2012.**  **The RCT reported March 2012** | **The CTR failed to automatically index RCT URL address or reference and the authors neglected to provide results.**  **The RCT reported results for the modified primary outcome and for secondary outcomes but failed to report that discharge at 12 hours was similar in both groups** | 11 |
| --- | --- | --- | --- | --- | --- | --- | --- |
| 15. NCT 01810978 https://clinicaltrials.gov (Treatment outcomes of infants with cyanotic congenital heart disease treated with synbiotics. Dilli et al. 132, 4, e932-e938 Oct 2013) | The CTR and RCT declared no funding source | The CTR and RCT declared identical sample size 100 infants > 35 weeks gestation up to 3 months with cyanotic congenital heart disease.  Setting: Neonatal Intensive Care Unit (NICU) Ankara, Turkey | **The CTR declared as inclusion criteria: infants > 35 weeks gestational age born or transferred to NICU with cyanotic congenital heart disease. Exclusion criteria congenital bowel anomalies.**  **The RCT reported as inclusion criteria: infants ≥ 35 weeks gestation parenterally-fed and survived beyond 7 days from admission. Exclusion criteria congenital bowel anomalies, and infants not parenterally-fed or died 7 days or more before admission (46 infants excluded because they died before 7 days of life. 8 refused to participate)** | **The CTR and RCT declared that the intervention group had been treated with bifidobacterium lactis plus inulin and control group with maltodextrin feeding.**  **The CTR declared sepsis as primary outcome and necrotising enterocolitis (NEC) as secondary outcome. The RCT reported nosocomial sepsis and NEC and secondary outcome length of stay in the NICU and death** | **The CTR declared completion date in May 2013.**  **The RCT reported Apr 2013** | **The CTR automatically indexed the RCT reference but the authors failed to report the results.**  **The RCT reported results from the upgraded secondary outcome: Bifidobacterium lactis administered parenterally decreased the incidence of nosocomial sepsis, NEC and death.**  *Enrolled infants between 2-10 days but excluded infants less than 7 days during the higher mortality rate* | 11 |
| 16. NTR 2061 and ACTRN 12610000230055 www.trialregister.nl www.anzctr.org.au (Mask versus nasal tube for stabilization of preterm infants at birth: a randomized controlled trial. Kamlin et al. 132, 2, e381-e388 Aug 2013) | **The CTR declared Australian National Health and Medical Research Council and Leiden University Medical Center.**  **The RCT reported Australian National Health and Medical Research Council and innovational research incentives scheme veni-vidi-vici** | **Two CTRs registered the study with different sample size: 774 preterm (25-30 weeks) eligible infants in the delivery room in the Netherlands Trial Register (NTR) and 648 in the Australian trial retrospectively registered in the Australian register (ANZCTR).**  **The RCT reported 648 preterm (24-29 weeks) eligible infants in the delivery room and 383 enrolled.**  **Settings: Leiden, (Netherlands) and Melbourne (Australia).**  **(Multicentre trial specified in the ANZCTR but not in the NTR. Registered retrospectively in the ANZCTR. In the NTR history viewable and trial status “stopped”. In the ANZCTR trial status “recruiting”** | **The NTR changed and updated inclusion criteria (28.10.2010: 24-week-old-infants will be included as well). The ANZCTR registered retrospectively, failed to report inclusion criteria changes** | Identical primary outcome in the CTR and RCT: mask vs nasal tube for intubation rate in preterm infants (24-30 weeks gestation) 24 hrs after birth | **Trial stopped on 05.04.2012 by a decision of the external data safety committee for slow recruitment and futility. The NTR reported reasons for early study stopping. The ANZCTR, failed to report trial stopping decision (online “still recruiting”)** | **The NTR, updated on 05.04.2012, failed to report completed results but stated that the trial was stopped for slow recruitment and futility. The ANZCTR was retrospectively registered and not updated (“still recruiting”).**  **The RCT reported no differences between intervention and control group but failed to report in the abstract the reasons why the trial stopped early. The RCT reported that in the nasal tube arm, data for 4 children were excluded from the analysis because nasal tube was unable to pass through the nostrils.**  *Intention-to-treat analysis not respected* | 11 |
| 17. CTRI/2010/091/001417 http://nims-icmr.nic.in (Short-course prophylactic zinc supplementation for diarrhea morbidity in infants of 6 to 11 months. Malik et al. 132, 1, e46-e52 July 2013) | **The CTR declared Indian Council medical research.**  **The RCT failed to report that trial was used for a postgraduate thesis (specified in the CTR)** | **The CTR sample size declared was 220 infants aged 6-11 months.**  **The RCT: sample size 272 infants 6-11 months.**  **Setting: New Delhi, India** | **The CTR failed to report inclusion from different settings.**  **The RCT reported that, to achieve the final sample size, additional children were recruited from a similar adjacent area (urban resettlement colony in Delhi India)** | **The CTR declared as primary outcomes: incidence of diarrhoea, acute respiratory tract infections and increase in height and weight in children aged 6-11 months enrolled over 5 months receiving zinc supplementation as a syrup.**  **The RCT reported as primary outcome: diarrhoea reduced by using zinc prophylaxis in children aged 6-11 months during a 1-year period.**  **(authors reported that they would publish results on additional outcomes separately** | Identical completion date January 2012 | **The CTR, updated by the authors on 14.06.2012, reported a brief summary of positive results for decreasing diarrhoea episodes and respiratory infections and increasing growth but failed to report side effects.**  **The RCT reported significant reduction in diarrhoea episodes at the end of 5 months of zinc supplementation.**  *Increasing side effects (vomiting and constipation) 11.9%-1.5% in the zinc group vs 4.8%-0% in the placebo group defined as not significant in the results and not reported in the abstract (reporting bias)* | 11 |
| 18. ACTRN 12612000976886 www.anzctr.org.au (Randomized controlled trial of a car safety seat insert to reduce hypoxia in term infants. McIntosh et al.132, 2, 326-331 Aug 2013) | The CTR and RCT declared same funding sources (University of Auckland, Research Foundation, and Cure Kids Charity | **The CTR failed to report sample size.**  **The RCT reported 80 enrolled infants (39 in the intervention and 41 in the control group).**  **Setting: University of Auckland, New Zealand** | The CTR and RCT declared to include healthy infants 5-11 days of life | **The CTR declared as primary outcome: number of oxygen desaturations measured by polysomnography during one daytime sleep period 30 min-3 hrs during sleep time during position in car seat at home.**  **The RCT reported as primary outcome: number of oxygen desaturations measured by polysomnography 30 min after the start of sleep and reassessment analysed in 30-minute intervals** | **The CTR was retrospectively registered (05.09.2012, four years after first participant enrolment 31.5.2008).**  **The RCT failed to provide completion date** | **The retrospectively registered CTR failed to index automatically the RCT URL address or reference and the authors neglected to provide results.**  **The RCT reported results for only one polysomnography lasting about 2 hours** | 11 |
| 19. ISRCTN03981121 www.controlled -trials.com (Randomized trial of a population-based, home-delivered intervention for preschool language delay. Wake et al.132, 4, e895-e904 Oct 2013) | National Health and Medical research council project grant application | **The CTR declared eligible 1500 4 years-aged children with language delay and 240 participants.**  **The RCT reported 1464 eligible pts and 200 participants.**  Setting: Single centre children’s hospital Melbourne Australia | The CTR declared that the study was started after two completed population-based trials and participants included in a large-scale single centre RCT nested within a cross-sectional study.  The RCT reported cross sectional with previous randomisation of two equally effective robust interventions (Let’s read and let’s learn language) | **The CTR declared primary outcome as expressive and receptive language at 5 and 6 years.**  **The RCT: expressive and receptive language at 5 years** | **The CTR declared completion date on March 2014.**  **The RCT failed to specify period of study conduction and reported results after 1 year instead of 2 follow-up years** | **The CTR automatically indexed the URL address of the published RCT, whereas the RCT neglected to report differences between the two arms of the study for the primary outcome.**  *The RCT reported a significant difference for a secondary outcome (phonological awareness skill).*  *High cost of speech therapy: 3231 Australian dollars per family* | 11 |

| 20. NCT 01604460 https://clinicaltrials.gov (Randomized trial of plastic bags to prevent term neonatal hypothermia in a resource-poor setting. Belsches et al.132,3, e656-e661 Sept 2013) | **The CTR declared funding from University teaching hospital Lusaka, Zambia and Alabama US. whereas the RCT reported grants from Eunice Kennedy Shriver Institute of Child Health** | The CTR and RCT reported the same sample size: 275 premature low-birth-weight and full-term infants.  Setting: multicentre trial University of Zambia, Africa and University of Alabama, US | **The CTR included infants within 10 minutes after birth and described the procedure after drying head.**  **The RCT included infants at the same time but without drying head** | **The CTR stated as primary outcome measure axillary temperature < 36.5 degrees Celsius (time frame 1-72 hours) using plastic bag within 10 min after birth in premature low-birth-weight and full-term infants.**  **The RCT reported axillary temperature > 36.5 degrees Celsius.**  *The temperature declared in the CTR (<36.5) is a probable typographic mistake* | **The CTR declared completion date on November 2012.**  **The RCT reported July 2012** | **The CTR automatically indexed the URL address of the published RCT, but the authors failed to report results.**  **The RCT neglected to report one hyperthermic infant in the intervention group in the abstract, table of results and conclusion** | 12 |
| --- | --- | --- | --- | --- | --- | --- | --- |

Two expert reviewers (PR and RD) identified and assessed the six reporting domains and, after several attempts, attributed the scores without taking into account small variations in the number of participants enrolled in the trial. Comments on inconsistencies made by the five investigator pairs are given in *Italics* and discrepant findings in bold print (higher discrepancy scores suggest risk of bias). CTR abbreviations: the United States National Institute of Health (NCT), the International Standard Randomised Controlled Trial Number (currently BioMed Central Open Access publishers) (ISRCTN), the Nederlands Trials Register (NTR), the Australian and New Zealand Clinical Trial registry (ACTRN), and the Clinical Trial Registry-India (CTRI). Clinical trials are listed according to the combined CTR-RCT discrepancy scores. When scores are identical the trials are listed alphabetically by first author surnames.
